# Preoperative muscle function assessments in nursing as predictors of postoperative outcomes in patients with colorectal cancer and malnutrition

**DOI:** 10.3389/fnut.2025.1559111

**Published:** 2025-04-11

**Authors:** Tingting Xie, Jiang Li, Xiaofen Wu, Xiangxiang Yang, Minmin Wang, Qiantong Dong, Xiaolei Chen, Weizhe Chen, Xiuyun Zheng

**Affiliations:** ^1^Department of Gastrointestinal Surgery Nursing Unit, Ward 442, The First Affiliated Hospital of Wenzhou Medical University, Wenzhou, China; ^2^Department of Gastrointestinal Surgery, The First Affiliated Hospital of Wenzhou Medical University, Wenzhou, China; ^3^Department of Nursing, The First Affiliated Hospital of Wenzhou Medical University, Wenzhou, China

**Keywords:** handgrip strength, gait speed, malnutrition, colorectal cancer, postoperative outcomes

## Abstract

**Background:**

Patients with colorectal cancer (CRC) often present with malnutrition upon admission, which is associated with a poor prognosis. However, current traditional tools for diagnosing malnutrition do not assess muscle function. This study aims to explore whether the evaluation of muscle function has predictive value for the prognosis of CRC patients with malnutrition.

**Methods:**

We retrospectively analyzed the clinical parameters of patients with CRC and malnutrition based on the Global Leadership Initiative on Malnutrition criteria who underwent radical surgery at our center from 2015 to 2021. Patients were grouped according to their preoperative muscle function assessments. The clinical characteristics and postoperative outcomes were compared between the groups. The risk factors for postoperative complications were analyzed.

**Results:**

A total of 346 patients were included in the study. Patients with low handgrip strength had higher incidences of total (*p* = 0.001), surgical (*p* = 0.015) and medical (*p* = 0.024) complications and longer postoperative hospital stays (*p* = 0.026). Patients with low gait speed had higher incidences of total (*p* = 0.027) and medical (*p* = 0.004) complications. Low handgrip strength was an independent risk factor for total (*p* = 0.008) complications, surgical (*p* = 0.038) complications and overall survival (*p* = 0.003). Low gait speed was an independent risk factor for medical complications (*p* = 0.021).

**Conclusion:**

For malnourished CRC patients, preoperative assessment of muscle function during perioperative care can predict adverse postoperative outcomes and can be incorporated into a comprehensive nutritional management plan.

## 1 Introduction

Malnutrition is a global public health concern. It is associated with a higher risk of infection, longer hospital stays, readmission, and higher costs. It is also considered an independent risk factor for mortality (1, 2). Cancer is a chronic wasting disease and the increased energy and protein requirements due to inflammation render patients susceptible to malnutrition (3). The prevalence of malnutrition in patients with cancer varies from 12% to 80%, depending on the diagnostic criteria and disease severity (4–6). Most patients with colorectal cancer (CRC) are malnourished at admission (7, 8). Nutritional assessment of hospitalized patients is crucial for early detection of malnutrition and timely initiation of nutritional therapy. The Global Leadership Initiative on Malnutrition (GLIM) developed diagnostic criteria for malnutrition in 2019 to improve global awareness of its diagnosis and treatment (9). Owing to the advantages of standardization, simplicity, and practicality, an increasing number of clinicians are using the GLIM criteria to diagnose malnutrition.

Most patients diagnosed with malnutrition according to the GLIM criteria suffer from loss of muscle mass, which often leads to loss of strength and functional impairment. However, the evaluation of muscle function is not included in the GLIM criteria. Experts recommend assessing muscle function as a supportive measure for the diagnosis of malnutrition (9). Moreover, the extent of the decline in muscle function is often more pronounced than that of muscle mass (10). Additionally, assessing muscle function is particularly important for the early detection of functional decline because impaired muscle function indicates the beginning of disability (11), which can further hinder anti-cancer treatment and lead to adverse clinical outcomes. Therefore, evaluating muscle function in patients at risk of malnutrition before surgery is a crucial preoperative assessment.

Many studies measure muscle function using handgrip strength (12). Low handgrip strength is a better predictor of short- and long-term mortality than low muscle mass (13) and is an independent indicator of malnutrition in patients with acute decompensated heart failure (14). Similarly, gait speed is increasingly being recognized as an indicator of lower limb muscle function. Gait speed is considered the “sixth vital sign” (15) and can be used to assess a person’s physical function level and risk of falls, serving as a reliable and sensitive measure of functional capacity. Low gait speed has been reported as an independent correlate of malnutrition risk in community-dwelling older adults (16), and there is a significant association between grip strength, gait speed, and risk of severe falls in this population (17). However, a recent literature review highlighted that physical activity, is not included in the tools for the early screening of malnutrition and/or malnutrition risk in CRC patients (18). Therefore, it remains unclear whether handgrip strength and gait speed are important independent predictors for patients with CRC and malnutrition.

Nutritional assessment has become an important component of the nutritional care for CRC patients. Several years ago, we incorporated preoperative handgrip strength and gait speed measurements into the perioperative nursing work and implemented them as part of the comprehensive nutritional management for CRC patients. The aim of this study is to investigate the predictive role of muscle function assessment in the prognosis of preoperatively malnourished CRC patients, and provide new insights and directions for improving the prognosis of these patients.

## 2 Materials and methods

### 2.1 Study population

This study retrospectively analyzed the clinical data of CRC patients who underwent radical surgery in the Department of Gastrointestinal Surgery of the First Affiliated Hospital of Wenzhou Medical University between February 2015 and October 2021. The inclusion criteria were as follows: (1) age over 18 years old, (2) confirmed by pathological examination to have CRC, (3) American Society of Anesthesiologists (ASA) grade ≤ III, (4) diagnosed as malnourished according to the GLIM criteria before surgery. The exclusion criteria were as follows: (1) emergency surgery, (2) other concomitant malignant tumors, (3) missing clinical data, and (4) pre-existing limb functional defects. This study was a part of a prospective study which was registered in the China Clinical Trial Registry (No. ChiCTR2200057818) and was approved by the Medical Ethics Committee of the First Affiliated Hospital of Wenzhou Medical University (No. 2015-023). All patients signed informed consent before surgery. This article has been reported in line with the STROBE guideline.

### 2.2 Clinical parameters and definitions

(1) Preoperative patient baseline characteristics: age, gender, body mass index (BMI), serum albumin concentration, hemoglobin concentration, Nutritional Risk Screening 2002 (NRS 2002) score, ASA grade, Charlson comorbidity index score, history of abdominal surgery, tumor-node-metastasis (TNM) stage, tumor location, third lumbar vertebra skeletal muscle index (L3-SMI), preoperative grip strength, preoperative gait speed; (2) Surgery-related data: surgical approach (laparoscopic or not), duration of surgery, combined organ resection; (3) Postoperative clinical outcomes, including postoperative complications within 30 days after surgery (graded according to Clavien-Dindo classification, this study only includes complications of grade II or above; complications of grade III or above are defined as severe complications), length of hospital stay, hospitalization costs, unplanned readmission within 30 days after discharge and overall survival. Surgical complications include: gastrointestinal dysfunction (including delayed gastric emptying and postoperative ileus or intestinal obstruction), incision complications, bleeding, seroperitoneum, anastomotic leakage; Medical complications include: pulmonary complications, cardiac complications, venous thrombosis, persistent hypoalbuminemia, and urological complications. Hypoalbuminemia is defined as serum albumin concentration < 35 g/L. Anemia is defined as hemoglobin concentration < 110 g/L for women and < 120 g/L for men.

### 2.3 Diagnosis of malnutrition

Hospitalized patients were diagnosed for malnutrition according to the GLIM criteria (9): First, we conducted NRS2002 nutritional risk screening to determine if the patient was at nutritional risk. Patients with an NRS2002 score of 3 or higher underwent further assessment. The diagnosis of malnutrition according to the GLIM involves combining at least one phenotypic criterion (involuntary weight loss, low BMI, or reduced muscle mass) and one etiological criterion (reduced food intake or absorption, disease burden, or inflammation). Upon admission, we asked the patients about their weight loss within the past 6 months. Weight loss is defined as ≥ 5% over the past 6 months, or ≥ 10% over more than 6 months. Muscle mass reduction was defined according to the L3-SMI, and we used previously studied cutoff values to define low SMI (19): < 34.9 cm^2^/m^2^ for women and < 40.8 cm^2^/m^2^ for men. Low BMI was defined as < 20 kg/m^2^ for individuals aged < 70, and < 18.52 kg/m^2^ for those aged ≥ 70. Because cancer is part of the etiological criteria of the GLIM standards, patients who met any of the phenotypic criteria were diagnosed with malnutrition.

### 2.4 Preoperative muscle function assessments and grouping

Since 2015, we have carried out assessments of muscle function in hospitalized patients, using tests for handgrip strength and gait speed immediately after admission. Handgrip strength data were obtained by measuring the dominant hand of the patient using an electronic handgrip dynamometer (EH101; CAMRY, South El Monte, CA). Patients were required to test their handgrip strength thrice, and the highest value of the three attempts was recorded as their handgrip strength. Low handgrip strength is defined as (20): < 18 kg for women and < 26 kg for men. The patients were then asked to walk at their normal pace over a distance of 6 m. Timing started when the patient took the first step and stopped when the first foot completely crossed the 6 m finish line. Three consecutive tests were performed, and the highest value was recorded for the patient’s gait speed. Low gait speed is defined as (20): < 0.8 m/s. We grouped patients preoperatively based on whether they had low handgrip strength or low gait speed.

### 2.5 Follow up

Patient received regular follow-up via telephone or outpatient visits after discharge, including monitoring of diet, physical condition, sleep quality, medication management, rehabilitation guidance, physical examination, and laboratory tests. We recommended follow-up visits every 3 months for the first 2 years postoperatively, and then every 6 months starting from the third year. The last follow-up was conducted on December 1st, 2023.

### 2.6 Statistical analysis

First, we tested whether the continuous data followed a normal distribution. Continuous data following a normal distribution are presented as mean and standard deviation, and an independent Student’s *t*-test was used to compare the two groups; for non-normally distributed continuous data, median and interquartile range were used, and a Mann-Whitney U test was used for comparison between groups. Categorical variables are described as the number of cases and percentages, and the chi-square test or Fisher’s exact test was used. Univariate and multivariate logistic regression analyses were used to identify independent risk factors for postoperative complications. Cox proportions hazards model analyses were used to identify independent risk factors for overall survival. Data analysis was performed using SPSS version 22.0 (IBM Corp, IBM SPSS Statistics for Windows, Armonk, NY) and MedCalc Software version 15.2 (MedCalc, Ostend, Belgium). Statistical significance was achieved when the two-tailed *p*-value was < 0.05.

## 3 Results

### 3.1 Population

A total of 346 patients were diagnosed with malnutrition and were included in this study. Among them there are 198 males and 148 females. The median age was 70 (range: 33–91) years old and the median BMI was 21.07 (range: 14.57–30.47) kg/m^2^. The median preoperative plasma albumin concentration of this study population was 37.3 (range: 22.7–53.4) g/L. The median preoperative hemoglobin concentration was 116 (range: 50–178) g/L. A total of 180 (52.0%) patients had comorbidities. A total of 83 (24.0%) patients had history of previous abdominal surgery. The median L3 SMI was 39.8 (range: 22.9–114.0) cm^2^/m^2^. The median handgrip strength was 22.0 (range: 4.8–50.2) kg. The median gait speed was 0.85 (range: 0.29–1.45) m/s. Laparoscopic surgery was performed in 147 (42.5%) patients.

### 3.2 Prevalence and characteristics of low handgrip strength and low gait speed in malnourished CRC patients

As shown in Table 1, 187 (54.0%) patients had low handgrip strength and 147 (42.5%) patients had low gait speed. In this study cohort, 114 (32.9%) patients had both conditions. The prevalence of low handgrip strength was 76.5% in the low gait speed population, while the prevalence of low gait speed was 61.0% in the low handgrip strength population.

**TABLE 1:** Demographic and clinical characteristic of patients.

Factors	Total (*n* = 346)	Handgrip strength		*P*	Gait speed		*P*	*P* ^a^
		Low (*n* = 187)	Normal (*n* = 159)		Low (*n* = 149)	Normal (*n* = 197)		
Age, median (IQR), years	70 (18)	76 (14)	64 (18)	< 0.001*	76 (12.6)	66 (18.5)	< 0.001*	0.837
Gender	–	–	–	0.001*	–	–	0.070	0.651
Male	198 (57.2)	92 (49.2)	106 (66.7)	–	77 (51.7)	121 (61.4)	–	–
Female	148 (42.8)	95 (50.8)	53 (33.3)	–	72 (48.3)	76 (38.6)	–	–
BMI, median (IQR), kg/m^2^	21.07 (4.21)	20.57 (4.59)	21.37 (3.86)	0.040*	20.57 (4.63)	21.16 (3.85)	0.188	0.843
Albumin, median (IQR), g/L	37.3 (6.2)	36.1 (5.8)	38.0 (6.8)	0.001*	35.8 (6.2)	37.9 (5.9)	< 0.001*	0.481
Hemoglobin, median (IQR), g/L	116 (30.3)	113 (34)	121 (29)	< 0.001*	111 (33.5)	119 (29.0)	< 0.001*	0.780
ASA grade	–	–	–	0.151	–	–	0.321	0.900
I	99 (28.6)	57 (30.5)	42 (26.4)	–	42 (28.2)	57 (28.9)	–	–
II	202 (58.4)	101 (54.0)	101 (63.5)	–	83 (55.7)	119 (60.4)	–	–
III	45 (13.0)	29 (15.5)	16 (10.1)	–	24 (16.1)	21 (10.7)	–	–
NRS 2002 scores, median (IQR)	4 (1)	3 (1)	4 (1)	0.608	4 (1)	4 (1)	0.851	0.906
Charlson comorbidity index	–	–	–	0.170	–	–	0.194	0.823
0	166 (48.0)	81 (43.3)	85 (53.5)	–	65 (43.6)	101 (51.3)	–	–
1	117 (33.8)	69 (36.9)	48 (30.1)	–	51 (34.2)	66 (33.5)	–	–
≥ 2	63 (18.2)	37 (19.8)	52 (16.4)	–	33 (22.1)	30 (15.2)	–	–
Previous abdominal surgery	83 (24.0)	46 (24.6)	26 (23.3)	0.773	40 (26.8)	43 (21.8)	0.279	0.639
L3 SMI, median (IQR), cm^2^/m^2^	39.8 (11.8)	36.9 (8.8)	43.5 (13.8)	< 0.001*	38.7 (12.6)	40.1 (12.0)	0.008*	0.146
Handgrip strength, median (IQR), kg	22.0 (13.4)	16.0 (8.1)	29.6 (8.8)	< 0.001*	16.8 (11.1)	25.0 (11.9)	< 0.001*	0.039*
Gait speed, mean (SD), m/s	0.85 (0.20)	0.77 (0.19)	0.94 (0.19)	< 0.001*	0.67 (0.11)	0.99 (0.13)	< 0.001*	< 0.001*
Tumor location	–	–	–	0.457	–	–	0.278	0.839
Right hemicolon	123 (35.5)	71 (38.0)	52 (32.7)	–	60 (40.3)	63 (32.0)	–	–
Left hemicolon	114 (33.0)	62 (33.2)	52 (32.7)	–	45 (30.2)	69 (35.0)	–	–
Rectum	109 (31.5)	54 (28.8)	55 (34.6)	–	44 (29.5)	65 (33.0)	–	–
TNM stage	–	–	–	0.066	–	–	0.680	0.866
I	66 (19.1)	27 (14.4)	39 (24.5)	–	25 (16.8)	41 (20.8)	–	–
II	142 (41.0)	80 (42.8)	62 (39.0)	–	66 (44.3)	76 (38.6)	–	–
III	136 (39.3)	78 (41.7)	58 (36.5)	–	57 (38.3)	79 (40.1)	–	–
IV	2 (0.6)	2 (1.1)	0 (0)	–	1 (0.7)	1 (0.5)	–	–

*^a^*Comparation between Low handgrip strength group and Low gait speed group.

*Statistically significant (*p* < 0.05). Values in parentheses are percentages unless indicated otherwise. IQR, interquartile range; SD, square deviation; BMI, body mass index; ASA, American Society of Anesthesiologists; L3 SMI, third lumbar vertebra skeletal muscle index; NRS, nutritional risk screening; TNM, tumor–node–metastasis.

Compared to patients with normal handgrip strength, those with low handgrip strength were older (*p* < 0.001), had a higher proportion of women (*p* = 0.001), lower BMI (*p* = 0.040), and lower preoperative serum albumin (*p* = 0.001) and hemoglobin levels (*p* < 0.001). Compared to patients with normal gait speed, the patients with low gait speed were older (*p* < 0.001) and, had lower preoperative serum albumin (*p* < 0.001) and hemoglobin levels (*p* < 0.001). Compared to the patients with low handgrip strength, those with low gait speed showed no significant differences in preoperative clinical characteristics.

### 3.3 Postoperative outcomes

**FIGURE 1:**
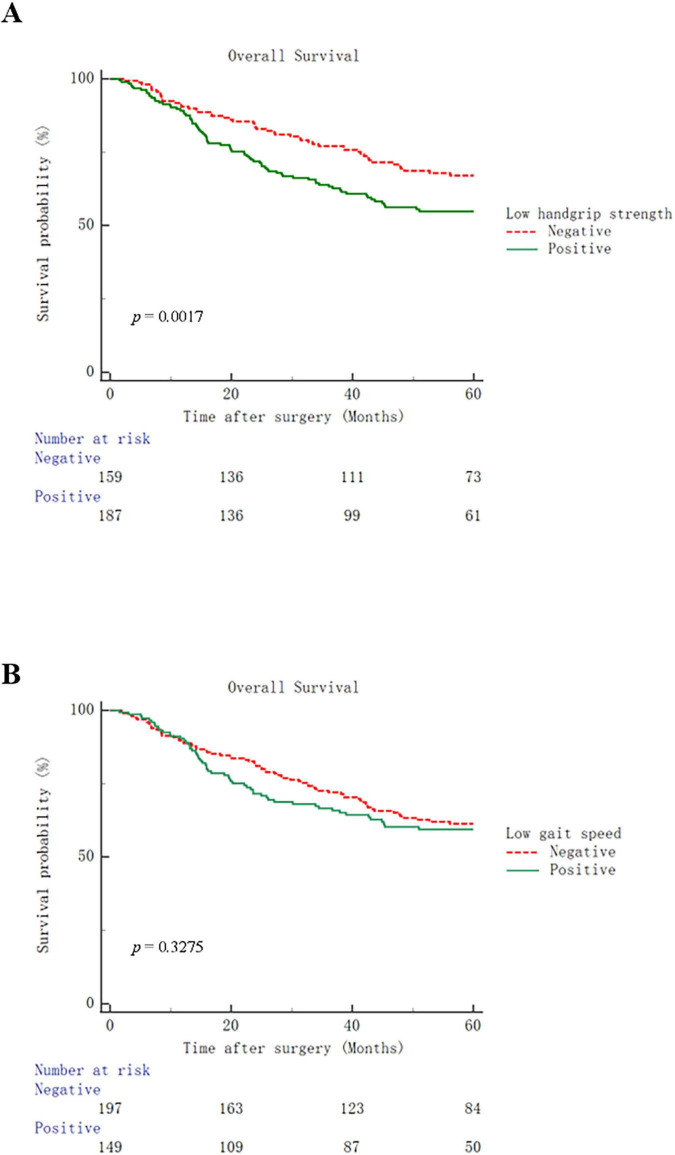
Kaplan–Meier curves for overall survival in patients with low handgrip strength **(A)** or low gait speed **(B)**.

The postoperative outcomes in this population are shown in Table 2. Total postoperative complications rate was 23.7% in all patients. The median postoperative hospital stay was 12 (range: 6–56) days. The incidences of total (30.5% vs 15.7%, *p* = 0.001), surgical (20.3% vs 10.7%, *p* = 0.015) and medical complications (12.8% vs 5.7%, *p* = 0.024) in patients with low handgrip strength were higher than those in patients with normal handgrip strength. In addition, patients with low handgrip strength tended to have longer postoperative hospital stays (median, 13 days vs. 12 days, *p* = 0.026) and higher hospitalization costs [median, 55201.3 yuan (7625.02 USD) vs. 49082.3 yuan (6779.79 USD), *p* = 0.002]. In the comparison grouped by gait speed, patients with low gait speed had higher incidences of total (29.5% vs 19.3%, *p* = 0.027) and medical complications (14.8% vs 5.6%, *p* = 0.004), and higher hospitalization costs [median, 54058.0 yuan (7467.09 USD) vs. 51795.9 yuan (7154.62 USD), *p* = 0.029]. Compared to patients with low handgrip strength, those with low gait speed showed no significant differences in postoperative outcomes. In terms of long-term outcome, compared with their respective control groups, patients with low handgrip strength had worse overall survival (*p* = 0.0017), while patients with low gait speed did not have this issue (Figure 1).

**TABLE 2:** Postoperative outcomes.

Factors	Total (*n* = 346)	Handgrip strength		*P*	Gait speed		*P*	*P* ^a^
		Low (*n =* 187)	Normal (*n =* 159)		Low (*n =* 149)	Normal (*n =* 197)		
Total complications	82 (23.7)	57 (30.5)	25 (15.7)	0.001*	44 (29.5)	38 (19.3)	0.027*	0.850
Surgical complications	55 (15.9)	38 (20.3)	17 (10.7)	0.015*	23 (15.4)	32 (16.2)	0.839	0.249
Medical complications	33 (6.6)	24 (12.8)	9 (5.7)	0.024*	22 (14.8)	11 (5.6)	0.004*	0.609
Severe complications	20 (5.8)	13 (7.0)	7 (4.4)	0.311	11 (7.4)	9 (4.6)	0.267	0.879
Surgical durations, median (IQR), minutes	167 (80)	170 (79)	164 (75)	0.328	167 (72)	167 (79.5)	0.610	0.457
Laparoscopic operation	147 (42.5)	84 (44.9)	63 (39.6)	0.321	66 (44.3)	81 (41.1)	0.554	0.909
Combined organ resection	27 (7.8)	16 (8.6)	11 (6.9)	0.571	17 (11.4)	10 (5.1)	0.030*	0.383
Postoperative hospital stays, median (IQR), days	12 (6)	13 (7)	12 (5)	0.026*	12 (5.5)	13 (6)	0.401	0.133
Hospitalization costs, median (IQR), yuan	53236.3 (21369.0)	55201.3 (22951.5)	49082.3 (20895.0)	0.002*	54058.0 (21958.4)	51795.9 (22782.6)	0.029*	0.876
Readmissions within 30 days of discharge	13 (3.8)	7 (3.7)	6 (3.8)	0.988	4 (2.7)	9 (4.6)	0.361	0.761

^a^Comparison between low handgrip strength group and low gait speed group.

*Statistically significant (*p* < 0.05). Values in parentheses are percentages unless indicated otherwise. IQR, interquartile range.

### 3.4 Analysis of risk factors for postoperative complications and overall survival

The factors associated with the overall postoperative complications are listed in Table 3. Univariate and multivariate logistic regression analyses revealed that hypoalbuminemia [Odds ratio 2.759 (1.631–4.666); *p* < 0.001], low handgrip strength [Odds ratio 2.085 (1.208–3.600); *p* = 0.008] and Charlson comorbidity index [≥ 2, Odds ratio 2.391 (1.217–4.697); *p* = 0.011] were independent risk factors for total postoperative complications. As for surgical complications, hypoalbuminemia [Odds ratio 2.624 (1.450–4.748); *p* = 0.001] and low handgrip strength [Odds ratio 1.941 (1.037–3.632); *p* = 0.038] were independent risk factors for surgical complications (Table 4). In terms of medical complications, hypoalbuminemia [Odds ratio 2.537 (1.177–5.470); *p* = 0.018], low gait speed [Odds ratio 2.533 (1.148–5.589); *p* = 0.021] and Charlson comorbidity index (≥ 2, Odds ratio 3.704 (1.500–9.151); *p* = 0.005] were independent risk factors for medical complications (Table 5). Lastly, hypoalbuminemia [Hazard ratio 1.649 (1.176–2.312); *p* = 0.004], low handgrip strength [Hazard ratio 1.685 (1.189–2.389); *p* = 0.003] and TNM stage [≥ III, Hazard 1.634 (1.168–2.285); *p* = 0.004] were showed to be independent risk factors for overall survival (Table 6).

**TABLE 3:** Univariate and multivariate logistic regression analysis of risk factors for total complications.

Factors	Univariate analysis		Multivariate analysis	
	Case with complication (%)	*p*	OR (95% CI)	*p*
Age	–	0.001*	–	–
≥ 65/< 65	67 (28.9)/15 (13.2)	–	–	–
Gender		0.455	–	–
Male/female	44 (22.2)/38 (25.7)	–	–	–
BMI	–	0.248	–	–
< 18.5	17 (24.3)	–	–	–
18.5–25	52 (21.8)	–	–	–
> 25	13 (34.2)	–	–	–
Hypoalbuminemia	–	< 0.001*	2.759 (1.631–4.666)	< 0.001*
Yes/no	45 (36.6)/37 (16.6)	–	–	–
Anemia	–	0.073		
Yes/no	45 (28.1)/37 (19.9)	–		
Low handgrip strength	–	0.001*	2.085 (1.208–3.600)	0.008*
Yes/no	57 (30.5)/25 (25.7)	–	–	–
Low gait speed	–	0.027*	–	–
Yes/no	44 (29.5)/38 (19.3)	–	–	–
ASA grade	–	0.045*	–	–
III/II, I	16 (35.6)/66 (21.9)	–	–	–
Charlson comorbidity index	–	0.018*	–	–
0	31 (18.7)	–	–	–
1	28 (23.9)	–	–	–
≥ 2	23 (36.5)	–	2.391 (1.217–4.697)	0.011*
Previous abdominal surgery	–	0.277	–	–
Yes/no	16 (19.3)/66 (25.1)	–	–	–
TNM stage	–	0.104		
I/II	43 (20.7)	–	–	–
III/IV	39 (28.3)	–	–	–
Combined resection		0.030*	–	–
Yes/no	11 (40.7)/71 (22.3)	–	–	–
Laparoscopic-assisted surgery	–	0.135	–	–
Yes/no	29 (19.7)/53 (26.6)	–	–	–
Tumor location	–	0.561	–	–
Rectum/colon	48 (22.6)/34 (25.4)	–	–	–

*Statistically significant (*p* < 0.05). OR, odds ratio; CI, confidence interval; BMI, body mass index; NRS, nutritional risk screening; ASA, American Society of Anesthesiologists; TNM, tumor–node–metastasis.

**TABLE 4:** Univariate and multivariate logistic regression analysis of risk factors for surgical complications.

Factors	Univariate analysis		Multivariate analysis	
	Case with complication (%)	*p*	OR (95% CI)	*p*
Age	–	0.011*	–	–
≥ 65/< 65	45 (19.4)/10 (8.8)	–	–	–
Gender		0.888	–	–
Male/female	31 (15.7)/24 (16.2)	–	–	–
BMI	–	0.650	–	–
< 18.5	11 (15.7)	–	–	–
18.5–25	36 (15.1)	–	–	–
> 25	8 (21.1)	–	–	–
Hypoalbuminemia	–	< 0.001*	2.624 (1.450–4.748)	0.001*
Yes/no	31 (25.6)/24 (10.8)	–	–	–
Anemia		0.449	–	–
Yes/no	28 (17.5)/27 (14.5)	–	–	–
Low handgrip strength	–	0.015*	1.941 (1.037–3.632)	0.038*
Yes/no	38 (20.3)/17 (10.7)	–	–	–
Low gait speed		0.839	–	–
Yes/no	23 (15.4)/32 (16.2)	–	–	–
ASA grade	–	0.093	–	–
III/II, I	11 (24.4)/44 (14.6)	–	–	–
Charlson comorbidity index	–	0.606	–	–
0	23 (13.9)	–	–	–
1	21 (17.9)	–	–	–
≥ 2	11 (17.5)	–	–	–
Previous abdominal surgery	–	0.681	–	–
Yes/no	12 (14.5)/43 (16.3)	–	–	–
TNM stage	–	0.069	–	–
I/II	27 (13.0)	–	–	–
III/IV	28 (20.3)	–	–	–
Combined resection	–	0.054	–	–
Yes/no	8 (29.6)/47 (14.7)	–	–	–
Laparoscopic-assisted surgery	–	0.317	–	–
Yes/no	20 (13.6)/35 (17.6)	–	–	–
Tumor location	–	0.608	–	–
Rectum/colon	32 (15.1)/23 (17.2)	–	–	–

*Statistically significant (*p* < 0.05). OR, odds ratio; CI, confidence interval; BMI, body mass index; NRS, nutritional risk screening; ASA, American Society of Anesthesiologists; TNM, tumor–node–metastasis.

**TABLE 5:** Univariate and multivariate logistic regression analysis of risk factors for medical complications.

Factors	Univariate analysis		Multivariate analysis	
	Case with complication (%)	*p*	OR (95% CI)	*p*
Age	–	–	–	–
≥ 65/< 65	28 (12.1)/5 (4.4)		–	–
Gender	–	–	–	–
Male/female	16 (8.1)/17 (11.5)		–	–
BMI	–	–	–	–
< 18.5	6 (8.6)		–	–
18.5–25	21 (8.8)		–	–
> 25	6 (15.8)		–	–
Hypoalbuminemia	–	–	2.537 (1.177–5.470)	0.018*
Yes/no	19 (15.4)/14 (6.3)	–	–	–
Anemia	–	–	–	–
Yes/no	20 (12.5)/13 (7.0)		–	–
Low handgrip strength	–	–	–	–
Yes/no	24 (12.8)/9 (5.7)		–	–
Low gait speed	–	–	2.533 (1.148–5.589)	0.021*
Yes/no	22 (14.8)/11 (5.6)	–	–	–
ASA grade		0.411	–	–
III/II, I	6 (13.3)/27 (9.0)		–	–
Charlson comorbidity index	–	–	–	–
0	11 (6.6)	–	–	–
1	9 (7.7)	–	–	–
≥ 2	13 (20.6)	–	3.704 (1.500–9.151)	0.005*
Previous abdominal surgery	–	–	–	–
Yes/no	4 (4.8)/29 (11.0)	–	–	–
TNM stage	–	–	–	–
I/II	18 (8.7)	–	–	–
III/IV	15 (10.9)	–	–	–
Combined resection	–	–	–	–
Yes/no	4 (14.8)/29 (9.1)		–	–
Laparoscopic-assisted surgery	–	–	–	–
Yes/no	11 (7.5)/22 (11.1)	–	–	–
Tumor location	–	–	–	–
Rectum/colon	19 (9.0)/14 (10.4)	–	–	–

*Statistically significant (*p* < 0.05). OR, odds ratio; CI, confidence interval; BMI, body mass index; NRS, nutritional risk screening; ASA, American Society of Anesthesiologists; TNM, tumor–node–metastasis.

**TABLE 6:** Univariate and multivariate logistic regression analysis of risk factors for overall survival.

Factors	Univariate analysis		Multivariate analysis	
	HR (95% CI)	*p*	HR (95%CI)	*p*
**Age**				
≥ 65/< 65	1.809 (1.223–2.676)	0.003*	–	–
**Gender**				
Male/female	0.876 (0.626–1.224)	0.438	–	–
**BMI**				
< 18.5/18.5–25	1.504 (1.018–2.222)	0.040*	–	–
> 25/18.5–25	0.771 (0.422–1.408)	0.397	–	–
Hypoalbuminemia	–	–	1.649 (1.176–2.312)	0.004*
Yes/no	1.711 (1.223–2.393)	0.002*	–	–
**Anemia**				
Yes/no	1.199 (0.859–1.675)	0.286	–	–
**Low handgrip strength**				
Yes/no	1.766 (1.214–2.568)	0.003*	1.685 (1.189–2.389)	0.003*
**Low gait speed**				
Yes/no	0.945 (0.657–1.359)	0.761	–	–
ASA grade				
III/II, I	1.574 (0.997–2.485)	0.051	–	–
**Charlson comorbidity index**				
1/0	1.211 (0.828–1.772)	0.324	–	–
≥ 2/0	1.541 (0.996–2.384)	0.052	–	–
**Previous abdominal surgery**				
Yes/no	0.953 (0.639–1.422)	0.814	–	–
TNM stage				
III–IV/I–II	1.574 (1.127–2.199)	0.008*	1.634 (1.168–2.285)	0.004*
**Combined resection**				
Yes/no	1.171 (0.632–2.170)	0.615	–	–
**Laparoscopic-assisted surgery**				
Yes/no	0.831 (0.589–1.173)	0.293	–	–
**Tumor location**				
Rectum/colon	0.697 (0.493–0.985)	0.041*	–	–

*Statistically significant (*p* < 0.05). HR, Hazard ratio; CI, confidence interval; BMI, body mass index; ASA, American Society of Anesthesiologists; TNM, tumor–node–metastasis.

## 4 Discussion

In this retrospective study, we observed that patients with CRC and malnutrition who were diagnosed with low handgrip strength or low gait speed had worse preoperative overall status. Patients with low handgrip strength had higher rates of overall, surgical, and medical complications, longer hospital stays and higher hospitalization costs. Patients with low gait speed had higher rates of overall and medical complications, and higher hospitalization costs. Low handgrip strength, hypoalbuminemia, and Charlson comorbidity index were independent risk factors for overall postoperative complications. Low handgrip strength and hypoalbuminemia were independent risk factors for surgical complications. Low gait speed, hypoalbuminemia, and Charlson comorbidity index were independent risk factors for medical complications.

Malnutrition is common among patients with CRC. In the present study, the prevalence of malnutrition was 24.7%. Malnutrition negatively affects postoperative complications, chemotherapy efficacy, and tolerance in CRC (8, 21). Therefore, nutritional assessments should be conducted to detect malnutrition and intervene early. In fact, malnutrition is a complex condition involving muscle loss, strength, and functional impairment, as well as reduced dietary intake and inflammation. This makes the determination using a single indicator difficult. Furthermore, a recent survey found that nearly 43% of clinicians, dieticians, and nurses lack confidence or the ability to identify malnutrition and sarcopenia (22). Currently, traditional tools for screening nutritional risk or malnutrition do not assess muscle function. The Subjective Global Assessment (SGA) and Patient-Generated Subjective Global Assessment (PG-SGA) mainly measure changes in muscle wasting, skin condition, and subcutaneous fat thickness. The Malnutrition Universal Screening Tool (MUST) assesses the patients’ nutritional status by considering BMI, weight changes, and disease impact. The Mini Nutritional Assessment-Short Form (MNA-SF) only confirms whether the patient is bedridden or able to go out. The GLIM criteria include only one phenotypic indicator related to muscle mass reduction. Malnutrition in patients with CRC is strongly associated with poor physical and mental health (23). Evaluating muscle strength is considered a good nutritional indicator, and assessing muscle function is a functional method for evaluating nutritional status. In times of malnutrition, skeletal muscle becomes the primary source of energy, leading to depletion of protein, muscle strength, and functional decline (24). Therefore, the assessment of muscle function may be a crucial addition to the overall evaluation of patients with malnutrition. In addition, decline in muscle strength is known to occur more rapidly than a decline in muscle mass (25). Muscle function assessment plays a role in the early identification of high-risk patients undergoing surgery.

In this study, 54.0% of the patients had low handgrip strength, 43.1% had low gait speed, and 32.9% had both conditions. Therefore, the prevalence of low handgrip strength is high in malnourished CRC patients. Interestingly, most patients diagnosed with low handgrip strength also had low gait speed. In terms of clinical characteristics, our study found that patients with low handgrip strength or low gait speed had poorer clinical features than the respective asymptomatic control groups. The main characteristic of patients with low handgrip strength was older age, lower BMI, and were a higher proportion of women, whereas the main characteristic of patients with low gait speed was older age. Due to the smaller body structure and muscle density in women, this physiological difference may contribute to the greater impact of malnutrition on handgrip strength (26). Furthermore, patients with either condition had lower serum albumin and hemoglobin levels. When comparing patients with low handgrip strength and low gait speed, we found no significant differences in preoperative clinical characteristics. This suggests that muscle dysfunction (whether low handgrip strength or low gait speed) and malnutrition have a synergistic effect, leading to a poorer overall preoperative status in patients with CRC and placing them in a vicious cycle. A recent study found that malnutrition was associated with more complex care needs in elderly residents with declining or frail physical function (27). This also indicates the need to provide more perioperative care and intervention for malnourished CRC patients with preoperative muscle dysfunction and to inform patients and their families about high-risk events during hospitalization in a timely manner.

For any muscle function assessment to be implemented in clinical practice, it must demonstrate the ability to identify high-risk individuals with adverse outcomes in malnourished patients. In terms of postoperative outcomes, our study found that patients with low handgrip strength had a significantly increased incidence of overall, surgical, and medical complications, as well as increased length of hospital stay and total costs, and worse overall survival. Patients with low gait speed had an increased incidence of overall and medical complications, as well as increased total costs. Previous research has shown that handgrip strength is significantly lower in hospitalized elderly patients with malnutrition as defined by the GLIM criteria (28). Handgrip strength is negatively correlated with the length of hospital stay, and is an independent predictor of hospital stay (29, 30). Furthermore, it has been shown to be positively correlated with cancer survival rates (31). Gait speed testing is commonly used to diagnose severe sarcopenia (32) and identify individuals at high risk of adverse health outcomes (33). Similar to handgrip strength, it is a simple and repeatable measure of physical performance. Both handgrip strength and gait speed reflect muscle function, and evidence suggests that the combined measurement of gait speed and handgrip strength is more sensitive than single characteristics or other possible combinations of dual factors (34). Therefore, we chose to simultaneously measure handgrip strength and gait speed in our nursing work. In clinical practice, we have also found that handgrip strength and gait speed are easy to measure, non-invasive, and can detect changes in muscle function status in patients with CRC and malnutrition before surgery. They can be repeatedly measured to provide real-time monitoring and guidance for postoperative care, and to focus on the occurrence of postoperative complications in malnourished CRC patients with low handgrip strength or gait speed. In the early postoperative period, assisting patients in changing positions and getting out of bed, as well as strengthening nutrition support strategies, are particularly important.

Multivariate analysis suggested that low handgrip strength is an independent risk factor for overall and surgical complications, and overall survival, while low gait speed is an independent risk factor for medical complications. This result indicates that these two can predict different aspects of clinical prognosis. The preoperative state of low handgrip strength indicates poorer tissue healing ability and more severe malnutrition in patients, leading to an increased incidence of postoperative complications and surgical complications. Patients with low gait speed are more likely to be less active in daily activities, and have poorer cardiorespiratory function and tolerance to Panza et al. (35). This increases the risk of falls after surgery and the probability of prolonged bed rest, preventing early postoperative ambulation and activity. It can also lead to postoperative medical complications such as cardiovascular disease, aspiration pneumonia, venous thrombosis, and urinary tract infections (36). This suggests that in postoperative care, it is crucial to focus different types of complications based on different types of muscle function decline, and appropriate recovery training should be provided. For patients with low handgrip strength, rehabilitation exercises, including active joint movement and strength training should be encouraged during the nursing process (37). For patients with low gait speed, moderate aerobic exercises such as walking and resistance training, can be encouraged to improve cardiorespiratory function and muscle endurance (38).

The strengths of this study include the first exploration of the prognostic value of preoperative muscle function assessment in predicting postoperative outcomes, specifically in malnourished CRC patients. However, this study has potential limitations. First, this was a retrospective, single-center study, and our findings should be interpreted with caution and validated in other populations. Second, we assessed handgrip strength in the dominant hand of patients. Recent research has indicated that handgrip strength asymmetry is associated with lower limb functional capacity and low gait speed (39), providing new avenues for future research. Lastly, although our study demonstrated that low handgrip strength and low gait speed increase the risk of adverse postoperative outcomes in malnourished CRC patients, the corresponding interventions and improvement measures were not explored. Other studies have suggested that exercise and appropriate nutritional therapy can improve handgrip strength (40), and future research should focus on this topic.

In conclusion, a preoperative assessment of muscle function has been carried out at our center, including tests for handgrip strength and gait speed. Handgrip strength and gait speed are easy to measure, non-invasive, can detect changes in muscle function status in malnourished CRC patients. Adding muscle function assessment to the perioperative care of malnourished CRC patients can predict adverse postoperative outcomes and survival. Preoperative muscle function assessment can serve as a supplementary evaluation of nutritional status and be incorporated into the comprehensive nutritional management plan for CRC patients. Future research needs to focus on how to improve malnourished CRC patients’ prognosis through muscle function.

## Data Availability

The raw data supporting the conclusions of this article will be made available by the authors, without undue reservation.
